# ROS1 Expression Correlates With Inguinal Lymph Node Affection in Vulvar Cancer Patients: A Retrospective Study

**DOI:** 10.1002/cam4.71160

**Published:** 2025-08-23

**Authors:** Meletios P. Nigdelis, Annick Bitterlich, Mariam Parvanta, Bashar Haj Hamoud, Erich Franz Solomayer, Martin Ertz, Laura Schnöder, Annette Hasenburg, Bernd Holleczek, Mathias Wagner, Gilbert Georg Klamminger

**Affiliations:** ^1^ Department of Gynecology, Obstetrics and Reproductive Medicine Saarland University Medical Center (UKS) Homburg Germany; ^2^ Department of General and Special Pathology Saarland University (USAAR) and Saarland University Medical Center (UKS) Homburg Germany; ^3^ Saarland University Medical Center for Tumor Diseases (UTS) Homburg Germany; ^4^ Department of Obstetrics and Gynecology University Medical Center of the Johannes Gutenberg University Mainz Mainz Germany; ^5^ Saarland Cancer Registry Saarbrücken Germany

**Keywords:** immunohistochemistry, ROS1, tyrosine kinase inhibitors, vulvar cancer

## Abstract

**Purpose:**

Systemic treatment options for vulvar squamous cell carcinoma (VSCC) are limited. ROS1, a tyrosine kinase implicated, for example, in non‐small cell lung cancer (NSCLC), has recently shown responsiveness to tyrosine kinase inhibitors. This study investigated immunohistochemical ROS1 expression in VSCC to explore its potential as a future therapeutic target in this rare malignancy.

**Methods:**

In this retrospective study, 48 patients with VSCC undergoing vulvectomy were included. Clinicopathological data were collected in a standardized manner. Immunohistochemistry (IHC) was used to assess ROS1 expression on an ordinal scale from 0 (absent staining) to 3 (> 50% of neoplastic cells demonstrated cytoplasmatic staining); levels 0 and 1 were considered negative, while 2 and 3 were rated as positive. After differences and correlations with clinicopathological parameters were evaluated between positive and negative tumors, we fitted logistic regression and survival models to assess the association of ROS1 with inguinal lymph node involvement and overall survival. Statistical analysis was conducted using GraphPad and Jamovi.

**Results:**

ROS1 IHC levels were associated with lymph node involvement [odds ratio (OR) 2.396, 95% confidence interval (CI) 1.034–5.555, logistic regression, *p* = 0.042]. ROS1 positive tumors demonstrated no difference in overall survival compared with negative ones [hazards ratio (HR) 0.837, 95% CI 0.283–2.479, log‐rank (Mantel‐Cox) test, *p* = 0.738].

**Conclusion:**

ROS1 expression was associated with inguinal lymph node involvement but not overall survival among VSCC patients. Further studies are required to elucidate the role of ROS1 in VSCC therapeutics.

## 1 Introduction

Vulvar cancer (VC) comprises the fourth most common gynecologic malignancy, with an increasing incidence of 6900 cases in the United States in 2024 and a significant mortality with almost 19,000 deaths worldwide in 2022 [1, 2]. The most common histologic subtype is squamous cell carcinoma, which has been subdivided into HPV‐dependent and HPV‐independent carcinomas based on p16 expression patterns on immunohistochemistry according to the most recent classification of the World Health Organization (WHO) [3]. HPV‐independent tumors typically develop in the context of chronic inflammatory conditions or dermatoses, most commonly lichen sclerosus. These tumors are often preceded by precursor lesions known as differentiated vulvar intraepithelial neoplasia (dVIN), which are often associated with alterations in the p53 tumor suppressor pathway [4]. Despite this diagnostic achievement in classification, few advancements have been made regarding the prognostic role of biomarkers and biomarker‐driven therapies, which are reserved only as second‐line treatments [5].

One of the recently studied molecular targets in oncology, especially glioblastoma and non‐small cell lung cancer (NSCLC), is ROS1. It constitutes a tyrosine kinase insulin receptor, encoded by the *ROS1* (alternatively c‐ros 1) proto‐oncogene (chromosomal region 6q22.1) [6, 7]. From a pathogenetic point, gene fusions between the 5′ of partner genes, including CD74, *EZR*, GOPC (depending on the neoplasm), and the 3′ regions of *ROS1* often play a pivotal role in the activation of cellular survival and growth signaling pathways, leading to oncogenesis [8]. On the other side, overexpression, splice variants, mutations, and amplifications have not been as strongly associated with carcinogenesis as fusions. In terms of therapeutics, ROS1 gene mutations constitute a target of recently approved tyrosine kinase receptor inhibitors, such as crizotinib, entrectinib, and ceritinib [8, 9].

The evidence linking squamous cell carcinoma of the vulva (VSCC) and ROS1 expression is scarce. From a pathophysiological standpoint, a potential role of ROS1 in HPV‐dependent carcinogenesis may be postulated, supported by experimental evidence demonstrating the regulation of ROS1 expression by HPV oncoproteins E6 and E7, observed in a variety of tumor entities including lung cancer [10, 11, 12]. Similarly, in terms of HPV‐independent tumors, alterations in the p53 pathway have been identified in ROS1‐mutated NSCLC, where they have been implicated in treatment resistance, prognosis, and tumor progression [13]. Although the precise etiopathogenic role of ROS1 pathway alterations leading to p53 alterations remains to be fully elucidated, such a mechanism may also be hypothesized in the context of VSCC.

In a recent genomic analysis of VC patients, Gordinier et al. demonstrated differential expression of ROS1 (based on whole genome and transcriptome sequencing) between VC cases with recurrence or mortality compared with VC cases without these outcomes. More specifically, the overexpression of ROS1 was associated with a worse clinical outcome [14]. This association has also been observed in other gynecologic malignancies, such as cervical adenocarcinoma, as demonstrated by Machida et al. in a cohort study involving 49 patients [15].

This retrospective study examined the clinical significance and prognostic value of ROS1 expression on immunohistochemistry in vulvar squamous cell carcinomas.

## 2 Materials and Methods

### 2.1 Study Cohort

This is a retrospective cohort study involving 48 patients undergoing vulvectomy for squamous cell carcinoma of the vulva before March 2024 (see bioethics committee approval). A detailed diagnostic pathological evaluation of the surgical specimen was conducted at the Saarland University Medical Center in Homburg, Germany. This evaluation was used to extract primary clinicopathologic data, including T‐Stage, N‐Stage based on the most recent TNM classification of vulvar cancer [16]. We conducted a patient record search using ICD‐O codes 8085/3, 8086/3, and 8070/3 in cooperation with the Saarland University Medical Center for Tumor Diseases (UTS) registry to identify eligible patients.

Patients were a priori excluded if (a) the surgical specimen would solely contain high‐grade squamous cell intraepithelial neoplasia (HSIL), (b) they had malignant tumors other than squamous cell carcinomas (e.g., adenocarcinomas, melanomas) c) or recurrent vulvar cancer cases, and (d) patients who underwent palliative surgery. Oncologic variables (i.e., local recurrences, development of metastases, survival) were provided by the “Saarland Cancer Registry,” a statewide‐operating registry.

### 2.2 Bioethics Committee Approval and Reporting Guidelines

The Ethics Committee of the state of Saarland (study identification number 249/23) gave approval on March 7, 2024. Patient data was handled according to the Declaration of Helsinki [17]. Patient consent was waived in accordance with the retrospective design of the study, as approved by the relevant ethics committee and in compliance with national legislation. The reporting of this study abides by the STrengthening the Reporting of OBservational studies in Epidemiology (STROBE) criteria (see Table S1).

### 2.3 Immunohistochemical Analyses and Interpretation

**FIGURE 1:**
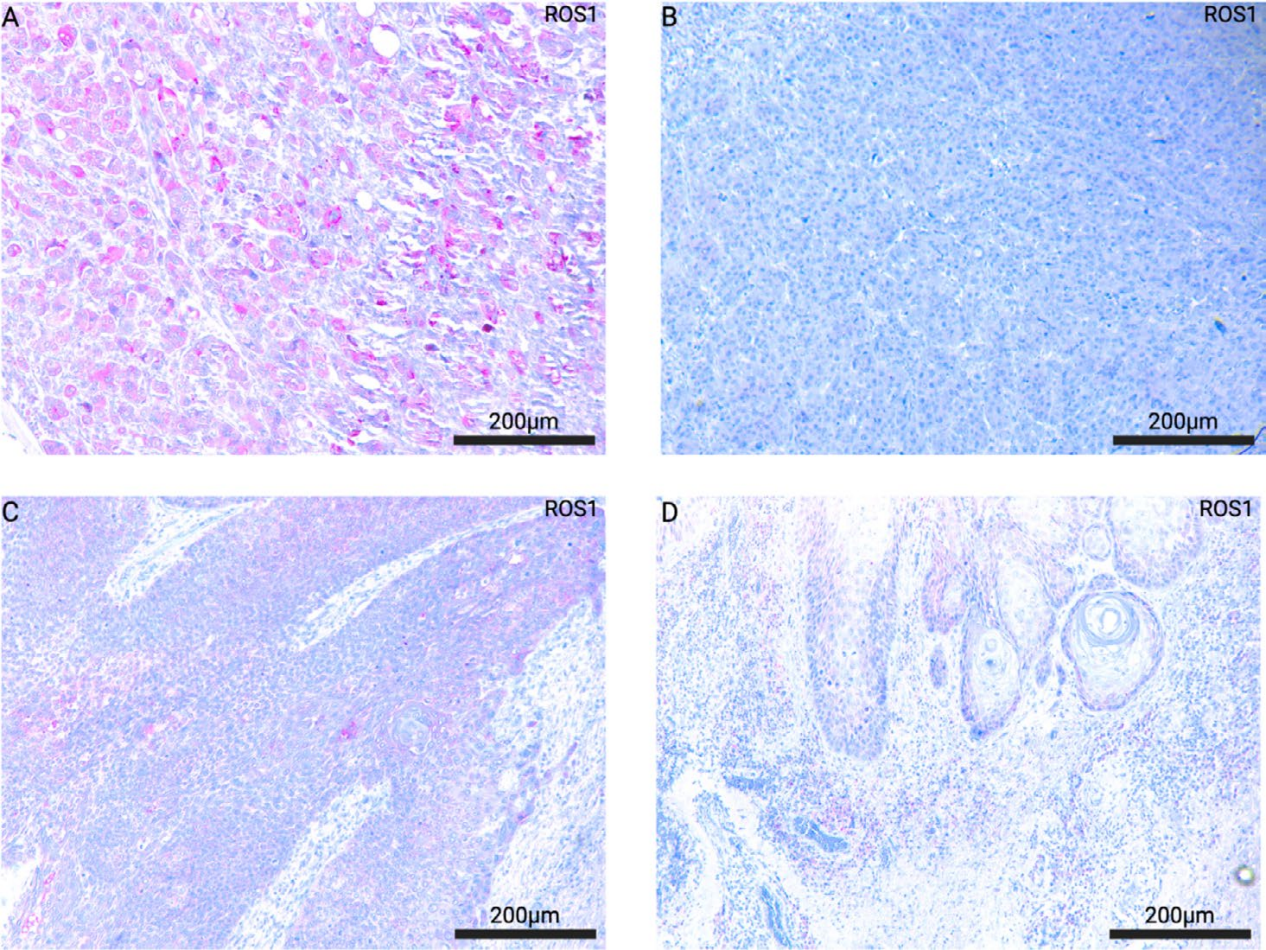
(A) Immunohistochemical ROS1 positive control (adenocarcinoma of the lung). (B) ROS1 negative VSCC without distinct areas of true staining. (C, D) Exemplary depiction of positive ROS 1 staining in VSCC. Positive neoplastic cells show a cytoplasmatic staining pattern.

Immunohistochemical analyses were conducted to examine patterns of expression of ROS1 in epithelial neoplastic cells. Hereby, corresponding slides of hematoxylin and eosin (H&E) stainings served as morphological control. A primary antibody for the ROS1 protein (clone: EP282, Medac GmbH/Epitomics, Wedel, Germany) was employed using the BenchMark ULTRA staining system in alignment with routine immunohistochemical diagnostic standards. We incorporated negative controls by leaving out the primary antibody, while ROS1 positive NSCLC tissue (adenocarcinoma of the lung) served as on‐slide positive control (see Figure 1).

In alignment with previous studies/standards [18], ROS1 expression was classified semiquantitatively: absent expression (0), single neoplastic cells with faint cytoplasmatic staining, comparable to background staining intensity (1), 0%–50% neoplastic cells with cytoplasmatic staining surpassing background staining intensity (2), 50%–100% neoplastic cells with cytoplasmatic staining surpassing background staining intensity (3). Apart from the ordinal classification, in this exploratory study tumors with staining patterns 0 or 1 were classified as “ROS1 negative” while tumors with staining patterns 2 or 3 were classified as “ROS1 positive,” in order to obtain a sufficient selectivity of true positive tumor cells.

As part of the study, p16 immunohistochemical staining of the corresponding diagnostic cases were systematically re‐evaluated. In accordance with criteria outlined in the “WHO Classification of Female Genital Tumors,” p16 was utilized as surrogate marker of HPV association. Tumors exhibiting a “block type” p16 expression pattern, characterized by at least 20 neighboring tumor cells with strong p16 staining were considered as HPV positive (HPV‐associated VSCC). In contrast, tumors displaying a “patchy” p16 expression pattern—defined by irregular nuclear and cytoplasmatic staining in a heterogenous distribution—or lacking p16 immunoreactivity were classified as HPV‐independent [3].

### 2.4 Statistical Analysis

Statistical analyses were conducted in GraphPad (Boston, MA 02110, US) and Jamovi [19] after all data were imported to a predefined Microsoft Excel file. Given the fact that no previous study on ROS1 IHC for VSCC has been published, we conducted no sample size estimation. Normality was assessed using the Shapiro–Wilk test. Summary statistics were generated for each variable. As none of the quantitative variables followed a normal distribution, we used median (interquartile range), while qualitative variables were reported as absolute frequency (percentage). We compared the clinicopathological characteristics of ROS1 negative and positive tumors using the Mann–Whitney test for continuous variables and the Chi‐squared or Fisher's exact test for qualitative variables. We used Spearman correlation analysis to assess the correlation between the intensity of ROS1 IHC with the clinicopathological parameters. We fitted a univariable logistic regression model between ROS1 expression and inguinal lymph node affection and constructed a receiver operating characteristic (ROC) curve to determine the area under the curve (AUC) of the predictive significance of ROS1 expression to determine inguinal lymph node involvement. Finally, we evaluated the effect of ROS1 expression status (positive versus negative) on overall survival using the log‐rank (Mantel‐Cox) test. Statistical significance was set as *p* < 0.05.

## 3 Results

### 3.1 Clinicopathologic Data

Table 1 summarizes the clinicopathological information of the patients in our study. A total of 48 patients with primary surgically treated VC with a median age of 62 years (interquartile range 46–75) were included in the analysis. Regarding T stage (according to the 2018 TNM‐guidelines, 8th edition), eight patients presented with T1a, 29 with T1b, 10 with T2, and 1 with T3. Nine patients demonstrated inguinal lymph node involvement (videlicet at least N1). After a median follow‐up of 44 months (interquartile range 12–135 months), 10 locoregional recurrences occurred; five patients experienced distant metastasis, and 17 (35%) patients died.

**TABLE 1:** Clinicopathologic characteristics of our cohort (*n* = 48).

Clinical parameter of interest	Value
Age	62 (46–75)
T‐Stage	
T1a	8 (16.7%)
T1b	29 (60.4%)
T2	10 (20.8%)
T3	1 (2.1%)
N‐Stage	
N0	39 (81.2%)
Positive groin lymph nodes (N1a/1b/2a/2b/2c)	9 (18.8%)
Infiltration depth (cm)	0.4 (0.1–1.0)
Lymphovascular infiltration (L1)	8 (16.6%)
Perineural infiltration (Pn1)	4 (8.3%)
Vascular infiltration (V1)	4 (8.3%)
Association with HPV	
HPV‐association VC	12 (25%)
HPV‐independent VC	36 (75%)
ROS1 positive	15 (31%)
Development of local recurrence	10 (20.8%)
Development of metastasis	5 (10.4%)
Follow‐up (months)	44 (12–135)

Abbreviations: HPV, human papilloma virus; VC, vulvar cancer.

### 3.2 Comparison of Clinicopathological Characteristics Between RO1 Negative and Positive Tumors

Table 2 demonstrates differences between the aforementioned clinicopathological parameters between ROS1 negative and positive tumors. Specifically, ROS1 positive tumors demonstrated higher rates of lymphovascular and perineural infiltration along with HPV independence compared with ROS1 negative ones. Still, these differences did not reach statistical significance (*p* > 0.05).

**TABLE 2:** Comparison of clinicopathological characteristics between ROS1 negative and positive tumors.

Clinical parameter of interest	ROS1 negative	ROS1 positive	*p*
Age	62 (49–72)	57 (45–79)	0.947^a^
T‐Stage			0.483^b^
T1a	6 (18.2%)	2 (13.3%)	
T1b	19 (57.6%)	10 (66.7%)	
T2	8 (24.2%)	2 (13.3%)	
T3	0	1 (6.7%)	
N‐Stage			0.115^b^
N0	29 (87.9%)	10 (66.7%)	
positive groin lymph nodes (N1a/1b/2a/2b/2c)	4 (12.1%)	5 (33.3%)	
Infiltration depth (cm)	0.4 (0.1–0.9)	0.6 (0.1–1.45)	0.655^a^
Lymphovascular infiltration (L1)	3 (9.1%)	5 (33.3%)	0.088^b^
Perineural infiltration (Pn1)	1 (3.0%)	3 (20.0%)	0.084^b^
Vascular infiltration (V1)	3 (9.1%)	1 (6.7%)	1.000^b^
HPV‐status			0.073^b^
HPV‐association VC	11 (33.3%)	1 (6.7%)	
HPV‐independent VC	22 (66.7%)	14 (93.3%)	
Development of local recurrence	5 (15.2%)	5 (33.3%)	0.151^c^
Development of metastasis	2 (6.1%)	3 (20.0%)	0.307^b^

^a^ *p*‐value corresponds to the Mann–Whitney *U* test.

^b^ *p*‐value corresponds to the Fisher's exact test.

^c^ *p*‐value corresponds to the Chi‐squared test.

### 3.3 Correlation of Classic Pathological Parameters With ROS1 IHC Expression Score

Spearmann correlation analysis between the ROS1 IHC score and pathological parameters failed to demonstrate significant associations between depth of infiltration, tumor stage, and vascular space invasion. On the contrary, significant associations were demonstrated with inguinal lymph node involvement, perineural infiltration, lymphovascular invasion, and HPV‐association. Table S2 demonstrates the results of Spearmann correlation along with two‐tailed *p*‐values.

### 3.4 Predictive Significance of the ROS1 IHC Score for Inguinal Lymph Node Affection

**FIGURE 2:**
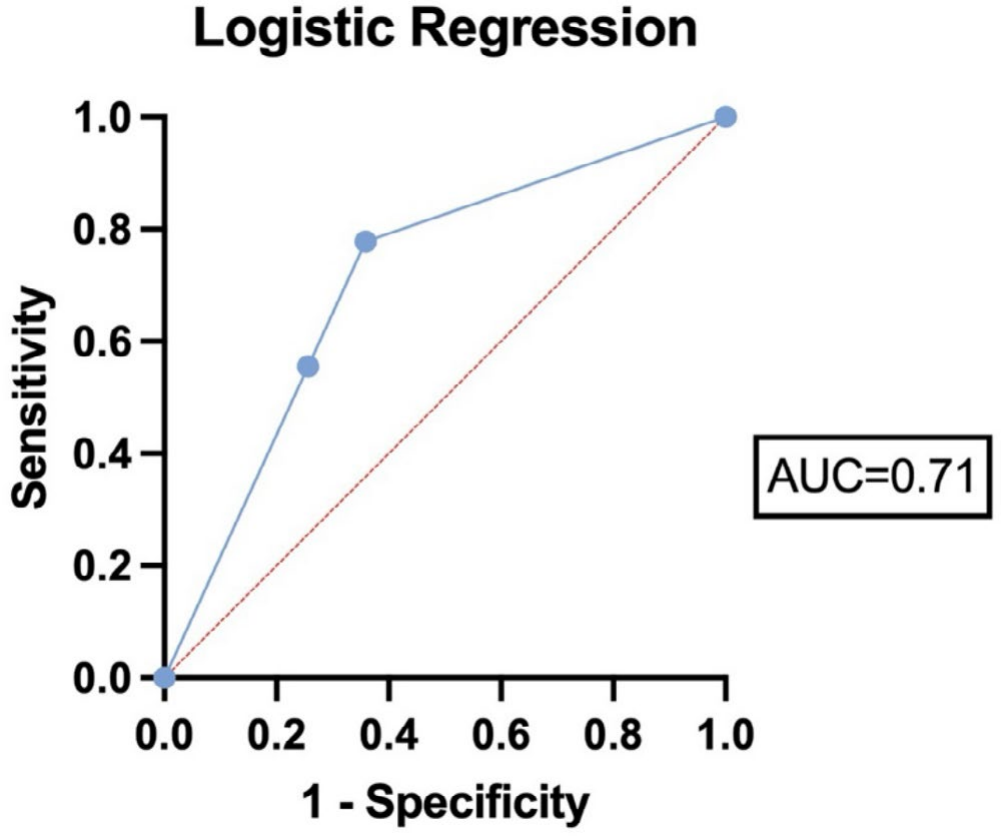
Receiver operating characteristic (ROC) curve evaluating the predictive significance of ROS1 IHC expression on inguinal lymph node involvement. The area under the curve (AUC) was 0.71, *p* = 0.052.

Simple logistic regression models between inguinal lymph node involvement and ROS1 IHC expression score yielded a positive significant association with an odds ratio (OR) of 2.396, 95% confidence interval (CI) 1.034–5.555 (*p* = 0.042). Based on this model, we constructed a ROC curve to predict the affection of inguinal lymph metastasis solely on ROS1 IHC expression (see Figure 2), with an AUC of 0.709, 95% CI 0.526–0.893, *p* = 0.052, a result trending toward significance.

### 3.5 The Impact of ROS1 Expression on Recurrence, Development of Metastasis, and Survival

**FIGURE 3:**
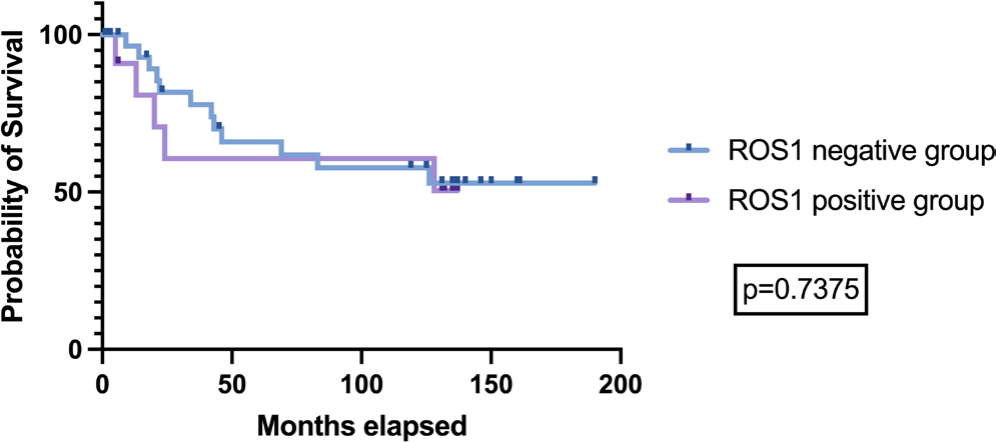
Kaplan–Meier curve on the effect of ROS1 expression status on overall survival. The *p*‐value corresponds to the log‐rank (Mantel‐Cox) test.

The development of local recurrence (*p* = 0.151; Chi‐squared test) or metastasis (*p* = 0.307; Fisher's exact test) was not statistically significant between ROS1 positive and negative tumors. After a median follow‐up of 44 months, there was no statistically significant difference in the overall survival between ROS1 positive and negative tumors [hazards ratio (HR) 0.837, 95% CI 0.283–2.479, log‐rank (Mantel‐Cox) test, *p* = 0.738, see Figure 3].

## 4 Discussion

This retrospective study demonstrated a significant correlation of ROS1 expression with perineural infiltration, lymphovascular infiltration, and HPV association. Importantly, ROS1 expression was significantly associated with inguinal lymph node involvement. Nevertheless, we could not demonstrate a significant difference in terms of local recurrence and overall survival between patients with ROS1‐positive tumors and those with ROS1‐negative tumors.

To our knowledge, this is the first exploratory study to evaluate ROS1 expression via IHC in the setting of VC. As a practical and widely adopted technique already integrated into the VSCC diagnostic workflow on a global scale, IHC allows for fast sample testing, even in resource‐limited laboratory settings; this potentially provides valuable preliminary evidence regarding the oncogenetic potential of specific protein expression patterns in VSCC cases. It should be noted that we have followed a standardized methodology (ordinal assessment of ROS1 expression) which has been consistently correlated with the presence of ROS1 fusions in lung cancer [8, 18]. Alternatives to our approach include fluorescence in situ hybridization (FISH) or molecular genetic techniques allowing for the exact characterization of ROS1 alterations, which fall outside the scope of our study.

Our findings differ from those of Gordinier et al., who identified a direct association between *ROS1* gene overexpression and local recurrence as well as mortality in vulvar cancer cases. Their study utilized next‐generation sequencing, whereas ours employed immunohistochemistry [14]. While we did not find a significant association between ROS1 expression, recurrence, and survival, its significant association with inguinal lymph node involvement suggests a potential surrogate marker. This finding warrants further investigation, as it aligns with the poorer prognosis observed in cases of ROS1 overexpression.

Another important observation requiring further study is the association with HPV status. Experimental evidence supports the hypothesis of ROS1 involvement in HPV‐dependent oncogenesis in two ways. First of all, both ROS1 and oncogenic E6/E7 proteins participate in the regulation of common signaling pathways (mostly affecting cellular proliferation and survival) such as the phosphatidylinositol 3‐kinase (PI3K)/Akt pathway [8, 20, 21]. Furthermore, it has been hypothesized that ROS1 transcription might be directly regulated by HPV oncoproteins, even though more studies are required for definitive proof of these biological processes [11].

Evidence on the pathognomic role of *ROS1* alterations (fusions, overexpression, or mutations) in other tumor types supports their potential as a prognostic and treatment target warranting investigation in gynecologic cancers as well. ROS1 fusions have been reported to affect different tumor types with different frequencies; for example, in the case of NSCLC, ROS1 fusions may be found in 1%–2% of the cases, while their involvement in other tumors, such as intrahepatic cholangiocarcinoma, seems more frequent, with frequencies reaching up to 37% [9, 22, 23]. In terms of prognosis, higher ROS1 expression has been shown to confer a protective prognostic effect in cases of intrahepatic cholangiocarcinoma and invasive ductal carcinoma of the breast [22, 24]. In terms of NSCLC, a large comparative study of different genetic alterations demonstrated a significant prognostic benefit in ROS1 mutated tumors compared with other gene mutations (e.g., *EGFR*, *KRAS*) [25].

From a therapeutic perspective, the development of tyrosine kinase inhibitors (TKIs) targeting ROS1 has significantly bolstered the treatment of ROS1 fusion‐positive tumors, particularly NSCLC. The first approved TKI, crizotinib, demonstrated high objective response rates (ORR) exceeding 65% and a median progression‐free survival (mPFS) of more than 15 months in most phase I and II clinical trials, bringing a substantial improvement over traditional systemic therapy options [26, 27, 28]. Nevertheless, the development of resistance after ROS1 TKIs is a common challenge, with mutations such as G2032R occurring in up to 41% of patients post‐crizotinib, which can hinder effective treatment [8]. Next‐generation TKIs, such as entrectinib and lorlatinib, aim to address resistance mechanisms, showing promising results, particularly in NSCLC patients with central nervous system (CNS) metastases [9]. The ongoing exploration of combination therapies and novel agents is essential for optimizing treatment strategies and improving patient outcomes in ROS1‐driven malignancies, including potential applications in other cancers like vulvar cancer.

The present study has limitations that should be taken into consideration when interpreting the findings. First of all, its retrospective and monocentric nature may constrain the ability of an a priori power calculation along with the external validity (generalizability) of the results, making future research in larger, prospective, multicentric cohort studies essential. Such attempts would further allow for multivariate testing and potentially strengthen our conclusions about the independent role of ROS1 in VSCC. Additionally, the reliance on IHC rather than molecular precision oncology techniques in this study poses a limitation in accurately characterizing *ROS1* fusions on a genomic level. We applied a reproducible IHC methodology enabling cost‐effective testing also in standard diagnostic‐laboratory settings. In our study, we chose a dichotomous evaluation approach, distinguishing between “true positive cells” and “negative/artificial staining.” From a biological perspective, this method is more appropriate for an exploratory design, which allows to reduce interpretative bias and focus on two distinct expression patters only. It should be noted that the lack of clearly defined and standardized cut‐off values in VSCC represents a broader methodological challenge, also affecting the interpretation of our results. Future studies should therefore aim to systematically evaluate and validate clinically relevant cut‐offs, ideally in large, well‐annotated cohorts and in correlation with patient outcomes.

In conclusion, we demonstrated a significant association between ROS1 expression and inguinal lymph node involvement. Although our study did not establish a significant association between ROS1 expression and overall survival, further research is warranted to elucidate the potential role of ROS1 fusions in the pathogenesis of squamous VC. Beyond its scientific relevance, the clinical applicability of TKIs remains a critical consideration, particularly given the limited availability of systemic treatment options.

## Author Contributions

**Meletios P. Nigdelis:** methodology; validation; data curation; formal analysis; writing – original draft; writing – review and editing. **Annick Bitterlich:** investigation; writing – review and editing. **Mariam Parvanta:** investigation; writing – review and editing. **Bashar Haj Hamoud:** investigation; writing – review and editing. **Erich Franz Solomayer:** investigation; writing – review and editing; resources. **Martin Ertz:** investigation; writing – review and editing; methodology; resources. **Laura Schnöder:** investigation; writing – review and editing. **Annette Hasenburg:** investigation; writing – review and editing; resources. **Bernd Holleczek:** investigation; writing – review and editing. **Mathias Wagner:** investigation; writing – review and editing; methodology; conceptualization; formal analysis; resources. **Gilbert Georg Klamminger:** conceptualization; methodology; supervision; writing – review and editing; writing – original draft; project administration.

## Ethics Statement

The study was conducted in accordance with the Declaration of Helsinki and approved by the Ethics Committee of Saarland (study identification number 249/23, approved on March 7, 2024).

## Conflicts of Interest

The authors declare no conflicts of interest related to this work.

## Supporting information


**Data S1:** cam471160‐sup‐0001‐DataS1.docx.

## Data Availability

Data insights that support the findings of this study are available on request from the corresponding author. The data are not publicly available due to privacy or ethical restrictions.
