# Supplementary material for: ROS1 Expression Correlates With Inguinal Lymph Node Affection in Vulvar Cancer Patients: A Retrospective Study
Source: Cancer Med. 2025 Aug 23;14(16):e71160. doi: 10.1002/cam4.71160 (PMC12374161; doi:10.1002/cam4.71160)
Supplement: Supplementary file 1 — Data S1: cam471160‐sup‐0001‐DataS1.docx. [file CAM4-14-e71160-s001.docx]

**Title:** ROS1 expression correlates with inguinal lymph node affection in squamous vulvar cancer patients: a retrospective study.

**Supplementary Table 1.** STROBE Statement—checklist of items that should be included in reports of observational studies.

**Note:** An Explanation and Elaboration article discusses each checklist item and gives methodological background and published examples of transparent reporting. The STROBE checklist is best used in conjunction with this article (freely available on the Web sites of PLoS Medicine at http://www.plosmedicine.org/, Annals of Internal Medicine at http://www.annals.org/, and Epidemiology at http://www.epidem.com/). Information on the STROBE Initiative is available at www.strobe-statement.org.

|  | Item No | Recommendation |
| --- | --- | --- |
| **Title and abstract** | 1 | (*a*) Indicate the study’s design with a commonly used term in the title or the abstract  **Response: See title and abstract (pp. 1-3)** |
|  |  | (*b*) Provide in the abstract an informative and balanced summary of what was done and what was found  **Response: this has been undertaken in the abstract (p. 3)** |
| Introduction | | |
| Background/rationale | 2 | Explain the scientific background and rationale for the investigation being reported  **Response: this has been undertaken as part of the introduction (pp. 4-5)** |
| Objectives | 3 | State specific objectives, including any prespecified hypotheses  **Response: page 4 (last sentence of the introduction)** |
| Methods | | |
| Study design | 4 | Present key elements of study design early in the paper  **Reponse: See Section 2. Materials and methods (pp. 5-8)** |
| Setting | 5 | Describe the setting, locations, and relevant dates, including periods of recruitment, exposure, follow-up, and data collection  **Reponse: See Section 2. Materials and methods (pp. 5-6)** |
| Participants | 6 | (*a*) *Cohort study*—Give the eligibility criteria, and the sources and methods of selection of participants. Describe methods of follow-up  *Case-control study*—Give the eligibility criteria, and the sources and methods of case ascertainment and control selection. Give the rationale for the choice of cases and controls  *Cross-sectional study*—Give the eligibility criteria, and the sources and methods of selection of participants  **Reponse: See Section 2. Materials and methods (pp. 5-8)** |
|  |  | (*b*) *Cohort study*—For matched studies, give matching criteria and number of exposed and unexposed  *Case-control study*—For matched studies, give matching criteria and the number of controls per case |
| Variables | 7 | Clearly define all outcomes, exposures, predictors, potential confounders, and effect modifiers. Give diagnostic criteria, if applicable  **Reponse: See Section 2. Materials and methods (pp. 5-8)** |
| Data sources/ measurement | 8* | For each variable of interest, give sources of data and details of methods of assessment (measurement). Describe comparability of assessment methods if there is more than one group  **Reponse: See Section 2. Materials and methods (pp. 5-8)** |
| Bias | 9 | Describe any efforts to address potential sources of bias  **Reponse: See Section 2. Materials and methods (pp. 5-8)** |
| Study size | 10 | Explain how the study size was arrived at  **Reponse: See Section 2.4. Statistical analysis** |
| Quantitative variables | 11 | Explain how quantitative variables were handled in the analyses. If applicable, describe which groupings were chosen and why  **Reponse: See Section 2.4. Statistical analysis** |
| Statistical methods | 12 | (*a*) Describe all statistical methods, including those used to control for confounding  **Reponse: See Section 2.4. Statistical analysis** |
|  |  | (*b*) Describe any methods used to examine subgroups and interactions |
|  |  | (*c*) Explain how missing data were addressed  **Reponse: See Section 2.4. Statistical analysis** |
|  |  | (*d*) *Cohort study*—If applicable, explain how loss to follow-up was addressed  *Case-control study*—If applicable, explain how matching of cases and controls was addressed  *Cross-sectional study*—If applicable, describe analytical methods taking account of sampling strategy |
|  |  | (*e*) Describe any sensitivity analyses  **Response: None undertaken** |

Continued on next page

| Results | | |
| --- | --- | --- |
| Participants | 13* | (a) Report numbers of individuals at each stage of study—eg numbers potentially eligible, examined for eligibility, confirmed eligible, included in the study, completing follow-up, and analysed  **Response: See Section 3.1.** |
|  |  | (b) Give reasons for non-participation at each stage |
|  |  | (c) Consider use of a flow diagram  **Reponse: Does not apply, given the exploratory nature of the paper.** |
| Descriptive data | 14* | (a) Give characteristics of study participants (eg demographic, clinical, social) and information on exposures and potential confounders  **Response: See Section 3.1.** |
|  |  | (b) Indicate number of participants with missing data for each variable of interest |
|  |  | (c) *Cohort study*—Summarise follow-up time (eg, average and total amount) |
| Outcome data | 15* | *Cohort study*—Report numbers of outcome events or summary measures over time |
|  |  | *Case-control study—*Report numbers in each exposure category, or summary measures of exposure  **Response: See Section 3.2.-3.5.** |
|  |  | *Cross-sectional study—*Report numbers of outcome events or summary measures |
| Main results | 16 | (*a*) Give unadjusted estimates and, if applicable, confounder-adjusted estimates and their precision (eg, 95% confidence interval). Make clear which confounders were adjusted for and why they were included  **Response: See Section 3.2.-3.5.** |
|  |  | (*b*) Report category boundaries when continuous variables were categorized |
|  |  | (*c*) If relevant, consider translating estimates of relative risk into absolute risk for a meaningful time period |
| Other analyses | 17 | Report other analyses done—eg analyses of subgroups and interactions, and sensitivity analyses |
| Discussion See pp. 10-14 (Discussion) | | |
| Key results | 18 | Summarise key results with reference to study objectives  **See pp 10-11** |
| Limitations | 19 | Discuss limitations of the study, taking into account sources of potential bias or imprecision. Discuss both direction and magnitude of any potential bias  **See pp 13-14** |
| Interpretation | 20 | Give a cautious overall interpretation of results considering objectives, limitations, multiplicity of analyses, results from similar studies, and other relevant evidence  **See pp 11-14** |
| Generalisability | 21 | Discuss the generalisability (external validity) of the study results  **See pp 13-14** |
| Other information | | |
| Funding | 22 | Give the source of funding and the role of the funders for the present study and, if applicable, for the original study on which the present article is based  **Does not apply** |

*Give information separately for cases and controls in case-control studies and, if applicable, for exposed and unexposed groups in cohort and cross-sectional studies.

**Supplementary Table 2.** Spearmann correlation analysis between ROS1 IHC score and pathological parameters.

| **Spearmann correlations:** ROS1 expression in association to | T-Stage | Infiltration depth | N | V | Pn | L | HPV-association |
| --- | --- | --- | --- | --- | --- | --- | --- |
| r | -0.001 | 0.119 | 0.309 | 0.017 | 0.340 | 0.307 | -0.322 |
| 95% confidence interval | -0.293 to 0.291 | -0.180 to 0.397 | 0.018 to 0.551 | -0.276 to 0,308 | 0.053 to 0.575 | 0.016 to 0.549 | -0.562 to -0.033 |
| Two-tailed p-value | 0.992 | 0.422 | 0.033 | 0.907 | 0.018 | 0.034 | 0.025 |

L: lymphovascular infiltration, N: inguinal lymphnode involvement, V: vascular infiltration, Pn: perineural infiltration
